# Process Oxygen Flow Influence on the Structural Properties of Thin Films Obtained by Co-Sputtering of (TeO_2_)_x_-ZnO and Au onto Si Substrates

**DOI:** 10.3390/nano10091863

**Published:** 2020-09-17

**Authors:** Leonardo Bontempo, Sebastião G. dos Santos Filho, Luciana R. P. Kassab

**Affiliations:** 1 Laboratório de Sistemas Integráveis, Escola Politécnica da Universidade de São Paulo, Av. Prof. Luciano Gualberto, 158, Travessa 3, 05508-900 São Paulo, SP, Brazil; sgsantos@usp.br; 2 Laboratório de Tecnologia em Materiais Fotônicos e Optoeletrônicos, Faculdade de Tecnologia de São Paulo, Centro Paula Souza, Praça Cel. Fernando Prestes, 30, 01124-060 São Paulo, SP, Brazil; kassablm@osite.com.br

**Keywords:** tellurate, gold nanoparticles, thin films, co-sputtering, TeO_2_ phases

## Abstract

In this study, we investigated the structural properties of TeO_2_-ZnO (TZ) and TeO_2_-ZnO-Au (TZA) thin films sputtered under different oxygen concentrations and either annealed or not annealed at 325 °C in air for 10 or 20 h. The lattice changes of the tellurium oxide were shown to be inherent in the polymorph properties of the α and β phases. The β phase was formed for null oxygen flow and the α phase was formed for different oxygen flows (0.5–7.0 sccm) during TZ and TZA sputtering. Au was encountered in its single phase or as AuTe_2_. The annealing had very little influence on the α and β phases for both TZ and TZA. It is worth noting that SiO_2_ and orthotellurate anions are both formed for not-null oxygen flow. An electrochemical mechanism was proposed to explain the SiO_2_ growth at the TZ/Si or TZA/Si interface, taking the orthotellurate anion as oxidizing agent into account.

## 1. Introduction

TeO_2_-ZnO (TZ) glasses exhibit interesting properties that make them attractive candidates for several applications involving nonlinear optics. Among oxi-tellurites, TZ glasses combine good mechanical stability, chemical durability, a high linear and nonlinear refractive index, low phonon energy (wavenumber around ∼ 750 cm^−1^) when compared to silicate and borate glasses, and a wide transmission window (0.4–6 μm). TZ glasses are capable of incorporating rare-earth ions and metallic nanoparticles (NPs) with an extensive range of photonic applications [1,2,3] and are potentially suitable materials for lasers and solar cells [4,5,6,7,8].

Some studies indicated that zinc tellurite bulk glasses (TeO_2_)_100-x_ (ZnO)_x_ (x = 0 to 10 wt%) form homogeneous solids and do not show phase separation [9,10], whereas the crystalline structure presents several polymorphous modifications in a variety of lattices in which the interatomic separation and interatomic bonding are slightly different [11]. Although the lattice polymorphisms are an intrinsic characteristic of TZ matrices, their interesting macroscopic properties have been advantageously employed as previously mentioned [1,2,3,4,5,6,7,8]. Another important application refers to the conduction and reversible memory phenomena of thin TeO_2_-ZnO-Au (TZA) films [12]. The main result was the uniform distribution of gold nanoparticles in the TZ matrix after co-sputtering of TZ and Au in Ar/O_2_ flow followed by annealing at 325 °C in air during 10 h. Although the matrix conditions for the reversible memory behavior have been well established, the influence of the oxygen flow during co-sputtering of TZ and Au onto silicon substrates on the lattice polymorphism as well as on the formation of different crystalline phases still requires further investigation.

At ambient conditions, TeO_2_ is reported to exist in two polymorphous forms, a yellow orthorhombic mineral tellurite, β-TeO_2_ [13,14], and a synthetic colorless paratellurite, α-TeO_2_ [14,15,16]. Most studies investigating the reaction chemistry were experiments involving paratellurite [17], which can be produced directly from the reaction of tellurium with oxygen (Te + O_2_ = TeO_2_) [14]. In addition, γ-TeO_2_ and δ-TeO_2_ were reported by Mirgorodsky et al. as other possible phases [11]; however, they were obtained in special conditions, i.e., by annealing pure TeO_2_ at 390°C during 24 h or by annealing a glassy sample of TeO_2_ containing 5–10 mol% of the modifier oxide WO_3_ during 24 h at 350 °C, respectively [11]. Additionally, the annealing at 800 °C in air of TeO_2_ modified with SB_2_O_5_ revealed γ-TeO_2_, α-TeO_2_, and SbTe_3_O_8_ [18].

In α-, β-, γ- and δ-TeO_2_ structures, tellurium atoms with oxidation state 4+ (Te^4+^) have four neighboring oxygen atoms and, in tellurate structures, tellurium atoms with oxidation state 6+ (Te^6+^) are rare in nature as tellurate anions are easily reduced to the tellurite anions [19,20,21], which makes the tellurate anion a possible oxidizing agent [22]. Only a few minerals with the tellurate anion have been discovered and many synthetic tellurates have been reported [21]. The spectroscopy of a range of tellurate anions has been reported with vibrational modes that are expected to occur, but not exclusively, in the 600–800 cm^−1^ region [21,22].

To shed further light on the influence of the co-sputtering oxygen flow on the lattice polymorphism and on the formed mixture of solid oxides, a physical characterization of gold and TeO_2_ crystalline phases as well as TZ-induced silicon oxide growth onto Si is presented in this work with the aid of Raman scattering analysis, transmission electron microscopy (TEM), RX and electron diffraction, and Rutherford backscattering (RBS) analysis.

## 2. Materials and Methods

The radio frequency (RF) magnetron co-sputtering method (13.56 MHz, PV300 model, Prest Vácuo, São Paulo, Brasil) was used to deposit TZ and TZA thin films onto silicon wafers with a 7.6 cm diameter and <100> crystallographic orientation with a resistivity ranging from 1 to 10 Ωcm. The conventional Radio Corporation of America (RCA) standard cleaning method [23] was used, followed by a final dip in a diluted hydrofluoric acid solution (d-HF) to clean the silicon wafers and to remove the thin surface oxide.

Ceramic targets fabricated from the starting powders of TeO_2_ and ZnO with a purity of 99.999% were mixed and then submitted to an eight ton uniaxial press, followed by sintering at 515 °C for 10 h. Targets with 5.0 cm diameter and 0.4 cm thickness were obtained with the following final stoichiometry: (TeO_2_)_3.3_-ZnO. Two targets were sputtered simultaneously for the production of the thin films with Au incorporation (TZA): the ceramic one (Target 1) presented above, and the gold one (commercial, Target 2), with a purity of 99.99%. Only Target 1 was sputtered for the production of the thin TZ films without Au.

Before film deposition, the base pressure was 6.7 × 10^−3^ Pa. Argon/oxygen plasma was used in the deposition process at 6.7 × 10^−1^ Pa (argon flow of 18 sccm and different oxygen flows of 0, 0.5, 1, 2, and 7 sccm for TZ and 0, 1, 2, 4, and 7 sccm for TZA). Target 1 was sputtered at 50W RF power to prevent damage and Target 2 at 6W RF. The substrate was maintained at a constant temperature during the deposition process (room temperature) and 15 cm away from the targets. TZ and TZA thin films at 100 nm thickness were produced with a deposition time of 75 min. TZ and TZA films were annealed at 325 °C in air for 10 and 20 h; thin films without annealing were also produced. Transmission electron microscopy (TEM) was used to determine the different crystalline phases along the film. The crystalline nature of the obtained structures could be determined employing electron diffraction analysis according to JCPDS-ICDD database [24]. For TEM measurements, the samples were milled, mixed with distilled water, and partially decanted. The floating part was taken using a metallic screen and analyzed by TEM.

Rutherford backscattering spectrometry (RBS) spectra were taken at 2.2 MeV under normal incidence of a ^4^He^+^ beam and with a scattering angle of 170° using a Pelletron Tandem accelerator, model 55 DH/NEC. The spectra were fitted with the aid of the SIMNRA 6.0 code [25] to obtain the Te, Zn, O, and Au aerial concentrations.

Raman spectra were recorded in 30–1100 cm^−1^ wavenumber using Alpha 330R model spectrometer (Witec, Ulm, Baden-Württemberg) with an Ar^+^ laser (514.5 nm, 150 mW) in a backscattering geometry to determine vibrational modes of TZ and TZA associated to the tellurium oxide, zinc oxide, and gold expected as deposited films [11,26]. The α and β phases of tellurium oxide were verified from Raman results by identifying the frequency modes as proposed by Mirgorodsky et al. [11] based on the almost exact matches for most of the vibrational frequencies and considering a maximum difference between the calculated and the measured frequency for each vibrational mode lower than 10% [11].

## 3. Results and Discussion

### 3.1. Structural Properties of TZ/Si

The structural properties of sputtered TZ on (100) silicon substrates were initially characterized considering the formation of the possible polymorphic phases of TeO_2_ and the observed formation of silicon oxide at the TZ/Si interface, which is a new important feature not reported previously and a possible mechanism formation is proposed based on Raman and RBS analyses.

**Figure 1:**
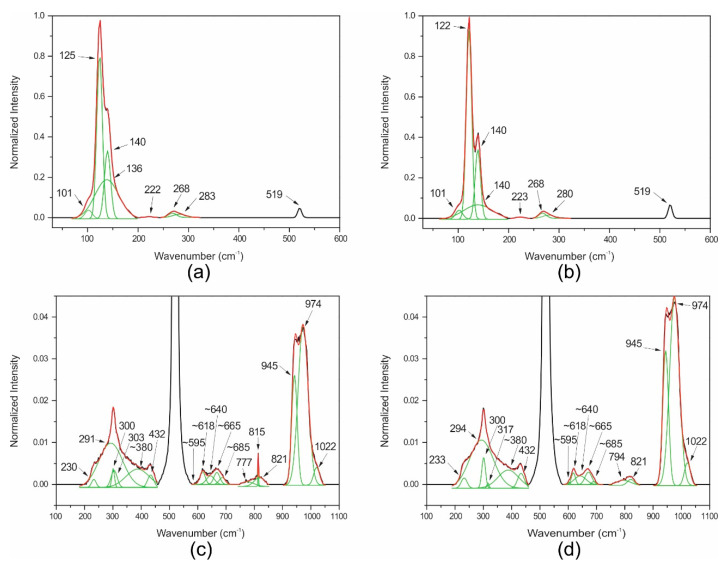
Measured Raman spectra (black line), fitted gaussian bands (green line) and sum of the fitted gaussian bands (red line) for TeO_2_-ZnO (TZ) thin films with different oxygen flow rates and annealing times: (**a**) 0 sccm/0 h; (**b**) 0 sccm/10 h; (**c**) 7 sccm/0 h; (**d**) 7 sccm/10 h.

Figure 1a–d show Raman results for TZ thin films with different oxygen flow rates and annealing times: (a) 0 sccm/0 h; (b) 0 sccm/10 h; (c) 7 sccm/0 h; (d) 7 sccm/10 h.

The deconvolution of each band allowed us to determine the different vibration modes, associated to the α and β phases of the tellurite oxide (presented in Table 1 [11,27]), using the criterion provided by Mirgorodsky et al. [11] as presented in Materials and Methods, to identify the vibration modes in the TZ films.

For null oxygen flow (Figure 1a,b), β was the detected phase (as indicated in Table 1) for both not annealed and annealed at 325°C in air. It is worth noting that the positions of the vibration frequencies barely change after annealing and most of them are located below 200 cm^−1^ as reported for the β phase [11].

For the 7 sccm oxygen flow (Figure 1c,d) cases, the positions of the vibrational frequencies were very different when compared with the null oxygen flow (Figure 1a,b). In this case, α was the formed phase detected (as indicated in Table 1) for both not annealed and annealed at 325 °C in air. We observed that the frequency positions almost do not change after annealing. It is worth noting that most of the frequency positions are located above 200 cm^−1^ as reported for the α phase [11] and the bending vibration mode of ZnO is 432 cm^−1^ [28].

In the range of 595 to 690 cm^−1^, two intense signals around 620 cm^−1^ and 667 cm^−1^ were observed with contributions of five different vibrational bands at 595 and 665 cm^−1^ assigned to α-TeO_2_, 618 and 685 cm^−1^, attributed to the Te^6+^O_6_ ν1 symmetric stretching and 640 cm^−1^ assigned to Te^6+^O_6_ ν3 antisymmetric stretching [22,29]. In addition, the large band at 380 cm^−1^ was assigned to Te^6+^O_6_ ν2 bending [29].

Thus, the film structure should contain both Te^4+^ and Te^6+^ with oxidation states of +4 and +6 for α-TeO_2_ and Te^6+^O_6_ anions, respectively. For null oxygen flow, only the β phase was observed and the Te^6+^O_6_^6−^ anions are not formed, which points out to a mechanism of orthotellurate (Te^6+^O_6_^6−^) anion formation assisted by the process oxygen during the film sputtering on Si substrates.

**Figure A1:**
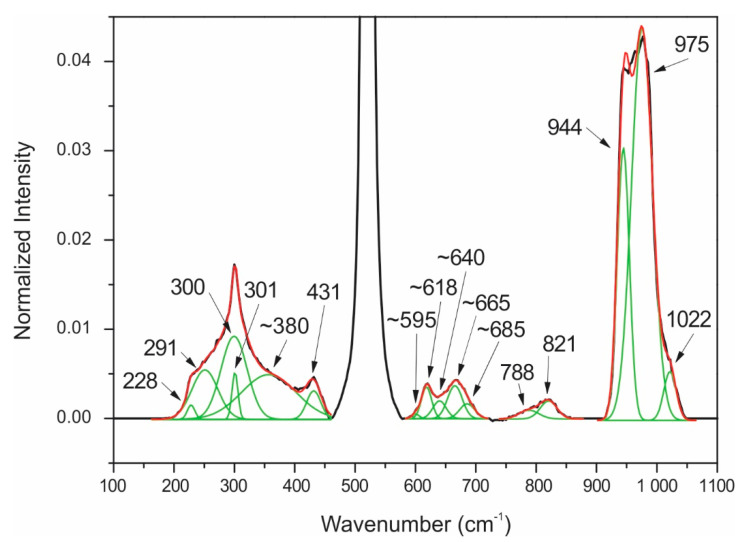
Measured Raman spectrum (black line), fitted gaussian bands (green line) and sum of the fitted gaussian bands (red line) for TZ thin films with process oxygen flow rate of 0.5 sccm without posterior annealing.

**Figure A2:**
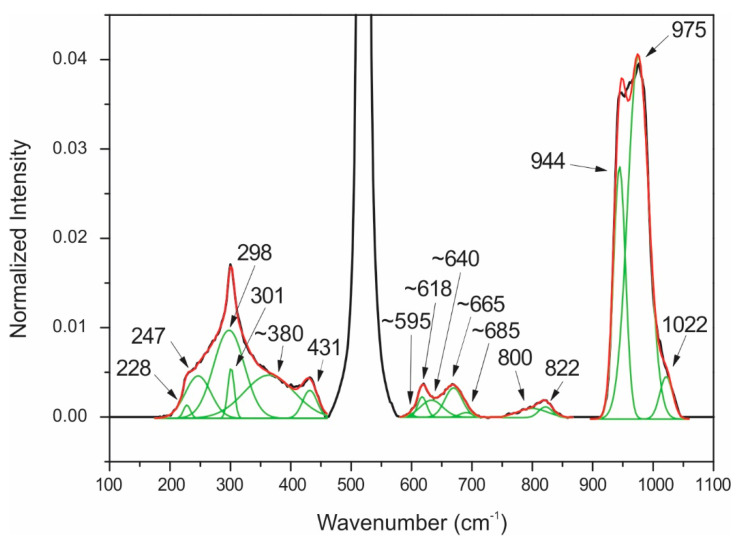
Measured Raman spectrum (black line), fitted gaussian bands (green line) and sum of the fitted gaussian bands (red line) for TZ thin films with process oxygen flow rate of 1 sccm without posterior annealing.

**Figure A3:**
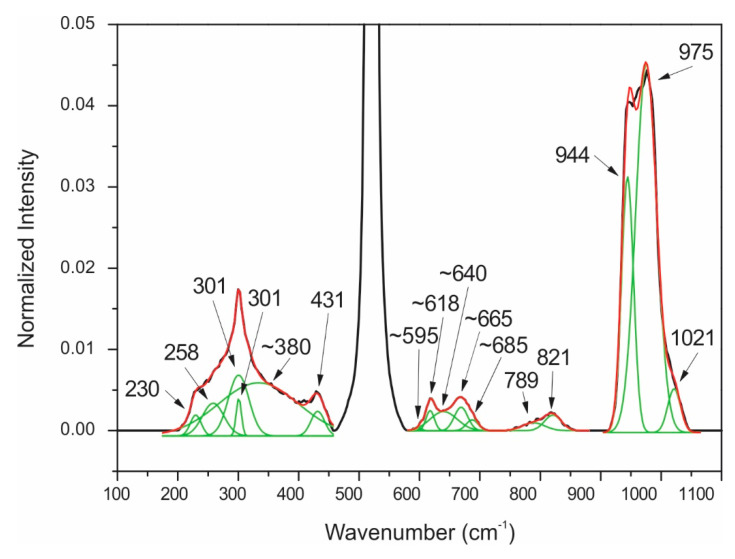
Measured Raman spectrum (black line), fitted gaussian bands (green line) and sum of the fitted gaussian bands (red line) for TZ thin films with process oxygen flow rate of 2 sccm without posterior annealing.

When above 800 cm^−1^ (shown in Figure 1c,d) we have the following vibration modes: Si-O stretching mode, metasilicate, Si-OH stretching mode, and Si-O-Si bending, which means the formation of silicon oxide during the TZ film deposition when oxygen is introduced (0.5–7 sccm) into the process chamber. The Raman spectra for oxygen flows below 7 sccm (see Figure A1, Figure A2 and Figure A3 in Appendix A) suggest an abrupt appearance of silicon oxide—that is to say, intense vibration modes associated with the silicon oxide in the Raman spectra are identified only for not-null oxygen flow. Since the deposited TZ films were around 100 nm thick, the contribution of silicon line 520 cm^−1^ to the measured spectrum becomes appreciable when SiO_2_ is formed [30] (as shown in Figure 1c,d). Literature corroborates the coexistence of separate phases of SiO_2_ and TeO_2_ at room temperature [31,32] for electrochemically induced sol–gel processes. To investigate further, we performed a quantitative analysis of the silicon oxide formation with the aid of RBS analysis.

**Figure 2:**
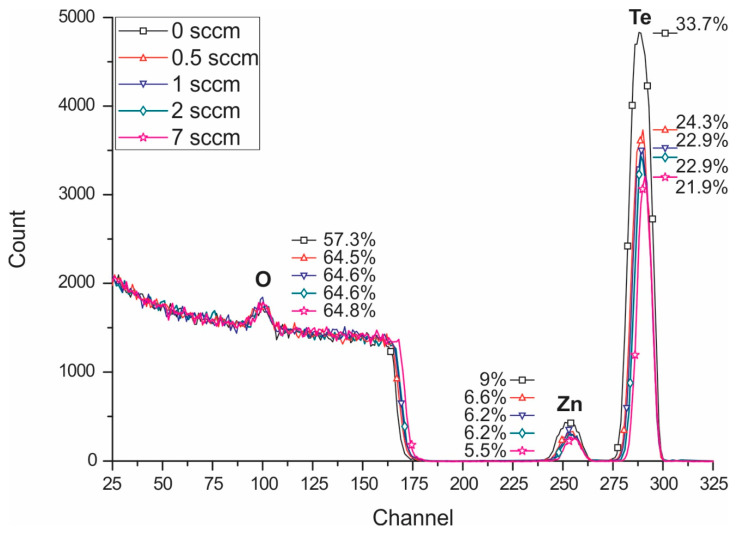
Rutherford backscattering spectrometry (RBS) results for TZ thin films for different oxygen flows: 0, 0.5, 1, 2, and 7 sccm (without annealing).

Figure 2 shows the RBS results for as-sputtered TZ thin films for different oxygen flows: 0, 0.5, 1, 2, and 7 sccm (without annealing). The spectra in Figure 2 were fitted with the aid of the SIMNRA 6.0 code [25] to obtain the Te, Zn, and O aerial concentrations. The abrupt decrease in the Te signal and the abrupt shift of the interface Si channel when the process oxygen flow increases from 0 to 0.5 sccm indicates two different behaviors—the conditions without and with process oxygen flows, respectively. These abrupt modifications in Figure 2 were understood as silicon oxide growth at the TZ/Si interface as pointed out by the RBS fitting and confirmed by Raman analysis.

The silicon oxide growth on silicon is reported to have a SiO_2_ stoichiometry for aerial concentrations higher than about 1 × 10^17^ cm^−2^ [33,34,35] according to the following chemical reaction: Si + O_2_ = SiO_2_.

Assuming SiO_2_ as the silicon oxide phase, a quantitative analysis of the different phases in the TZ film was performed with the aid of RBS analysis to obtain the total aerial concentration (cm^−2^) of TeO_2_, ZnO, and SiO_2_. The percentage values indicated in Figure 2 are relative to the total aerial concentration modeled as the sum of two different aerial concentrations of two layers: the first one containing TeZn_p_O_q_ = kTeO_2_ + lZnO and the second one containing TeZn_x_Si_y_O_z_ = mTeO_2_ + nZnO + oSiO_2_, which is essentially silicon oxide in the TeO_2_-ZnO structure due to the already observed reaction between the Si substrate and the oxygen during the sputtering process. This two-layer modeling allowed for a fine fitting of the RBS spectra for each TZ film deposited with different oxygen flows (0, 0.5, 1, 2, and 7 sccm), which pointed out to buried silicon oxide at the TZ/Si interface.

**Figure 3:**
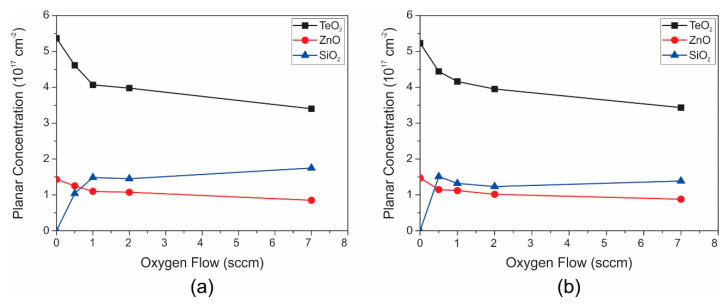
Planar concentrations (cm^−2^) for TeO_2_, ZnO, and SiO_2_, taken from the RBS study, as a function of the oxygen flow: (**a**) without annealing and (**b**) with annealing.

Figure 3a,b show the obtained aerial concentrations as a function of the oxygen flow for annealed and not annealed samples, respectively. As predicted by Raman analysis, the null oxygen flow means the null concentration of silicon oxide and not-null oxygen flow means aerial concentrations of silicon oxide in the percentage range.

In Figure 3a, one can highlight a silicon-oxide aerial concentration that gradually increases until about 1.47 × 10^17^ cm^−2^ when the process oxygen flow varies from 0 to 1 sccm and it slightly increases up to 1.75 × 10^17^ cm^−2^ for process oxygen flow in the range of 1 to 7 sccm, which was attributed to the decrease in the oxygen diffusion as the TZ aerial concentration increases making the surface more filled and closed. It is important to point out that this growth mechanism occurs at room temperature and is assisted by molecular oxygen introduced during the sputtering process.

On the other hand, comparing the not annealed (Figure 3a) with the samples annealed in air at 325 °C (Figure 3b), a slight peak of SiO_2_ for low oxygen flows occurs, indicating additional silicon oxidation, possibly, as already mentioned before, due to molecular oxygen introduced during the annealing in air at 325 °C.

The encountered planar concentration of ZnO in the film is next to 1/5 of the concentration used in the target for sputtering. This lower concentration of ZnO in the film was attributed to the volatilization of Zn during the sputtering process.

The SiO_2_ growth at room temperature can be understood as an electrochemical process on the silicon surface since tellurate is an oxidizing agent and can be easily reduced to tellurite as evidenced by the reported standard reduction potential (E° ~ −1.0 V) [22]. The proposed electrochemical reaction can be represented by the possible generic reduction and oxidation (redox) reactions as follows:

2Te^6+^O_6_^6−^ + 12e → 2Te^4+^O_3_^2−^ + 3O_2_ (reduction), (1)

[(2Si) = Si_S_^2−^]_3_ + 3O_2_ → (2Si_S_^2−^)_3_ + 3SiO_2_ + 12e (oxidation), (2)

(2Si) = Si^2−^ + 2Te^6+^O_6_^6−^ → (2Si^2−^)_3_ + 3SiO_2_ + 2Te^4+^O_3_^2−^ (redox), (3)

where (2Si) = Si_S_^2−^ is a pictorial representation for a <100> silicon substrate containing surface silicon Si_S_ with two surface bonds and two subjacent atoms (2Si), Equation 1 represents the reduction of the orthotellurate anions to tellurite, Equation 2 represents the oxidation of the surface silicon atoms to grow SiO_2_, and Equation 3 represents the overall redox reaction given by the sum of the reduction and oxidation reactions.

The proposed electrochemical mechanism takes into account the observed SiO_2_ growth at the TZ/Si interface, the silicon consumption as the SiO_2_ grows, the process activation by molecular oxygen introduced during the sputtering process and the formation of tellurite that is incorporated in the α-TeO_2_ structure. In addition, the aerial concentration of SiO_2_ starts saturating for higher process oxygen flow rates because reaction 2 became limited by the oxygen diffusion through the TZ and SiO_2_ as they are formed.

Based on the proposed model, a question which arises refers to how tellurates anions is in the matrix structure considering the formation of SiO_2_ and α-TeO_2_. Unfortunately, there exist very few works relating SiO_2_ and tellurates together. However, the existing literature [36,37] points out the possible formation of a three-dimensional anionic network of {Zn_6_[TeO_6_][Si_2_O_7_]_2_}^6−^ built up from one-dimensional chains of [Zn_6_TeO_18_]^18−^ interconnected by [Si_2_O_7_]^6−^ and packed with cations [36,37].

### 3.2. Structural Properties of TZA/Si

The structural properties of the sputtered TZA on (100) silicon substrates were also characterized. In this case, silicon oxide was formed at the TZA/Si with similar aerial concentrations as those shown in Figure 3 as a function of the oxygen flow rate. In addition, the aerial concentration of gold slightly decreased from ~8.0 × 10^16^ cm^−2^ to ~6.4 × 10^16^ cm^−2^ when the oxygen flow rate was varied in the range of 1 to 7 sccm, which was also a similar behavior observed for TeO_2_, ZnO, and SiO_2_ (see Figure 3), possibly because the quantity of charged metal oxide species that are pumped out of the sputtering chamber increases when the process oxygen flow rate is increased.

**Figure 4:**
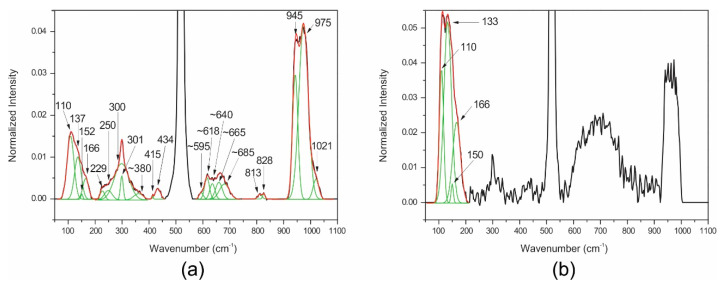
Measured Raman spectra (black line), fitted Gaussian bands (green line) and sum of the fitted gaussian bands (red line) for TeO_2_-ZnO-Au (TZA) thin films with 7 sccm oxygen flow and different annealing time: (**a**) 0 h; (**b**) 10 h.

As in the case of TZ, the silicon oxide at the TZA/Si was not observed for the null process oxygen flow in the Raman analysis. Figure 4a,b shows the Raman results for TZA thin films with 7 sccm oxygen flow and annealing time of 0 h and 10 h, respectively. The incorporation of Au significantly modifies the Raman spectra that now have frequency modes at 110, 137, 150, and 166 cm^-1^ of the compound AuTe_2_ [38], and the α phase of the TZ matrix was identified using almost exact matches for the most of the vibrational frequencies, as described in Materials and Methods. These results are presented in Table 2.

As in the case of TZ, two intense signals around 620 cm^−1^ and 667 cm^−1^ were also observed in the range of 595 to 690 cm^−1^, indicating that the co-sputtering of gold did not have an appreciable influence on the formed phases. In the range of 595 to 690 cm^−1^, the contribution of the same five vibrational bands were assigned at 595 and 665 cm^−1^ for α-TeO_2_, 618 and 685 cm^−1^ for Te^6+^O_6_ ν1 symmetric stretching, and 640 cm^−1^ for Te^6+^O_6_ ν3 antisymmetric stretching [22,29]. In addition, the large band at 380 cm^−1^ was assigned to Te^6+^O_6_ ν2 bending [29].

**Figure A4:**
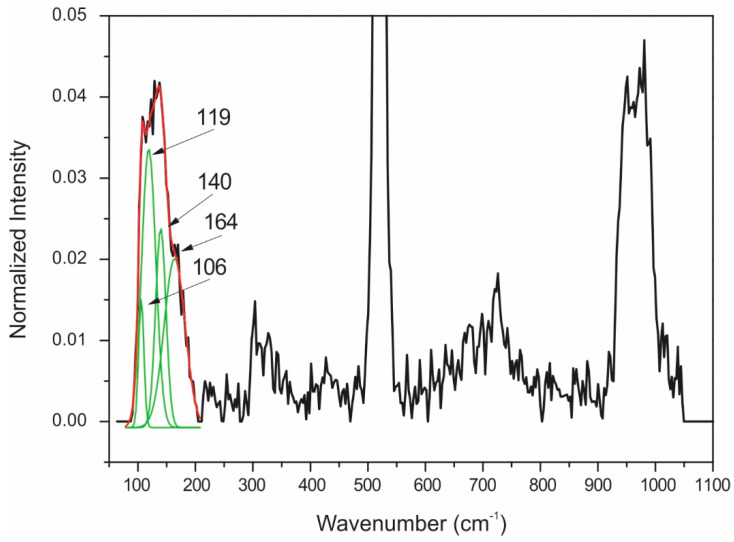
Measured Raman spectrum (black line), fitted gaussian bands (green line) and sum of the fitted gaussian bands (red line) for TZA thin films with process oxygen flow rate of 1 sccm and posterior annealing at 325 °C in air for 10 h.

Comparing Figure 4a,b, one observes the appearance of noisy bands above 200 cm^−1^ due to the formation of a homogeneous distribution of nanoparticles [12] after 10 h of annealing (Figure 7) that lowers the Raman scattering [39] and whose composition is Au and/or AuTe_2_, as Table 2 and Table 3 show (see also Figure A3 and Figure A4 in Appendix A for process oxygen flow rates of 2 and 4 sccm, respectively). This scattering lowering effect in the Raman spectra that diminishes the sensitivity for vibrational frequencies associated with the α phase of the tellurium oxide indirectly corroborates a greater sensitivity for the positions below 200 cm^−1^, where the vibrational frequencies of AuTe_2_ are directly observed.

Above 800 cm^−1^, the following vibration modes were also observed: Si-O stretching mode, metasilicate, Si-OH stretching mode, and Si-O-Si bending, which also means that the formation of silicon oxide occurred during the TZA film deposition when oxygen was introduced (0.5–7 sccm).

To confirm AuTe_2_, ZnO and α-TeO_2_ phase formation, TEM analysis was performed for different process oxygen flows of 1 sccm, 4 sccm, and 7 sccm. The results obtained from electron diffraction measurements were used to determine the crystalline structures for the case of 1 sccm oxygen flow. For 4 sccm and 7 sccm oxygen flows, the crystalline structures were determined directly from TEM images, as will be presented.

**Figure 5:**
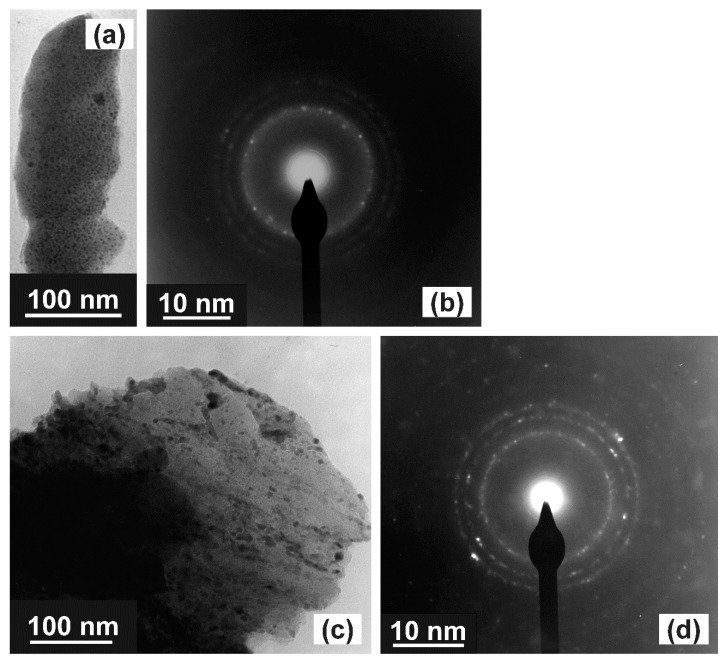
TEM image and electron diffraction for TZA thin films with 1 sccm oxygen flow and annealing time: (**a**) and (**b**) 10 h; (**c**) and (**d**) 20 h.

Figure 5a,c shows TEM images and Figure 5b,d shows the electron diffraction pattern for 1 sccm oxygen flow and after annealing of 10 and 20 h at 325 °C in air, respectively. In Figure 5a,c, we see that the darker dots in TEM images are related to Au-compound nanoparticles, whereas the clearer regions are related to the TZ matrix.

Figure 5b,d presents the polycrystalline diffraction rings obtained from electron diffraction measurements that correspond to the reflection from the crystalline planes that are used to calculate the interplanar distances (d_spacing_) and the corresponding crystalline structures, presented in Table 3, obtained by comparison with the JCPDS-ICDD database [24]. In this case, we observe the formation of Au, AuTe_2_, ZnO, and α-TeO_2_ crystalline phases. We recall the absence of β-TeO_2_ crystalline phase; the same behavior was observed for TZ thin film as Raman results showed the formation of β-TeO_2_ crystalline phase only for null oxygen flow. With the increase in the annealing time from 10 to 20 h, multiple crystalline facets at different interplanar distances (d_spacing_) appear, and, in most cases, it is not possible to distinguish Au, AuTe_2_, ZnO, and α-TeO_2_ phases due to the interplanar distances superposition (see Table 3). Individual facets exist for each one of these phases, which means it is highly probable they are present in the film, as can be seen by the results obtained from Raman measurements, shown in Table 2.

The present electron diffraction measurements results for 1 sccm oxygen flow complement those obtained from Raman, performed for 7 sccm oxygen flow (Figure 1 and Table 2). Moreover, the annealing time increase also favors the formation of Au in crystalline form, as can be seen by the growth of the diffraction rings concentration and the interplanar distances (d_spacing_), presented in Figure 5d and Table 3, respectively.

**Figure 6:**
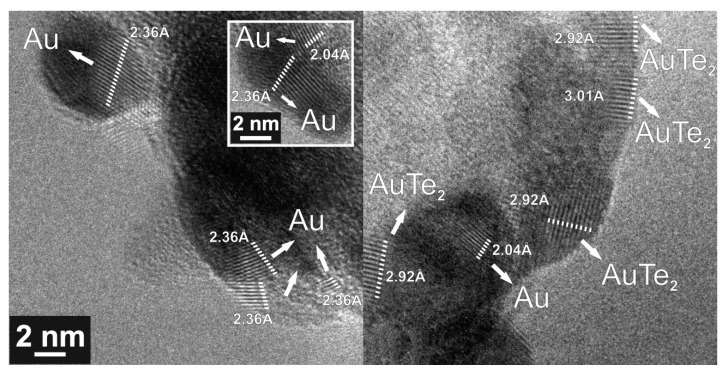
TEM image for TZA thin films with 4 sccm oxygen flow without annealing.

**Figure 7:**
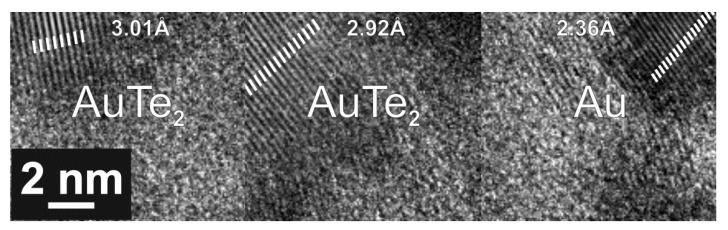
TEM image for TZA thin films with 7 sccm oxygen flow annealed during 10 h.

Since the results for oxygen flow higher than 1 sccm are similar to those obtained in Table 3, the following figures (Figure 6 and Figure 7) only show the top view TEM and present the interplanar distances (d_spacing_) ascribed to Au and AuTe_2_ crystalline phases obtained from a direct measurement method. Figure 6 shows TEM results for TZA with 4 sccm oxygen flow without annealing. It is possible to observe that gold is preferably hosted in darker regions (TeO_2_) that correspond to Au or AuTe_2_ phases, even for thin films without annealing, as can be seen by the large presence of crystalline planes shown in Figure 6. The same applies to 7 sccm oxygen flow, annealed for 10 h, as illustrated in Figure 7. We highlight that the presence of AuTe_2_ could also be observed in Raman measurements, for 7 sccm oxygen flow and 10h annealing, as shown in Table 2. Moreover, the different interplanar distances (d_spacing_) ascribed to Au (2.0 Å and 2.36 Å) and AuTe_2_ (2.92 Å and 3.01 Å) are attributed to different crystalline plane reflections related to Au ((hkl) = (200) and (hkl) = (111)) and AuTe_2_ ((hkl) = (201) and (hkl) = (11−1)) crystalline phases [24,40].

For the observed AuTe_2_ phase, a melting point around 460 °C (9000 J/mol), reported in reference [40], points out to a lower forming temperature. We can assume that the formation of AuTe_2_ is possibly eased by the presence of oxygen during the sputtering process.

## 4. Conclusions

TeO_2_-ZnO (TZ) and TeO_2_-ZnO-Au (TZA) thin films were prepared using the sputtering technique with different oxygen flows, without annealing and with annealing at 325 °C, in air, for 10 and 20 h.

TeO_2_ was detected in two polymorphous forms: both α and β phases. The β phase was formed for null oxygen flow and the α phase was formed for different oxygen flow (0.5–7.0 sccm) for TZ and TZA films. Au was encountered either in its pure crystalline phase or as the AuTe_2_ compound. The later annealing at 325 °C had very little influence on the TeO_2_ phase formation for both TZ and TZA.

Finally, it is worth noting that silicon oxide was found to grow for not-null oxygen flow, and from RBS analysis, it was found at TZ/Si and TZA/Si interfaces. We found that silicon oxide growth occurs at room temperature assisted by molecular oxygen introduced during the sputtering process and it was attributed to an electrochemical process on the silicon surface where orthotellurate anions act as oxidizing agent to grow SiO_2_ at the TZ/Si or TZA/Si interface. The proposed electrochemical mechanism also takes into account the silicon consumption as the SiO_2_ grows, the process activation by molecular oxygen introduced during the sputtering process, and the formation of tellurite that is incorporated in the α-TeO_2_ structure.

## Figures and Tables

**Table 1:** Raman results for TZ thin films with different oxygen flow rates and annealing times.

**0 sccm ^1^ 0 h**		**0 sccm 10 h**	
101	β ^2^	101	β
125	β	122	β
136	β		
140	β	140	β
222	β	223	β
268	β	268	β
283	β	280	β
520	Silicon line	520	Silicon line
**7 sccm 0 h**		**7 sccm 10 h**	
230	α ^3^	233	α
291	α	294	α
300	α	300	α
303	α	317	α
~380	Te^6+^O_6_ ν2 bending	~380	Te^6+^O_6_ ν2 bending
432	Zn-O bending	432	Zn-O bending
520	Silicon line	520	Silicon line
~595	α	~595	α
~618	Te^6+^O_6_ ν1 symmetric stretching	~618	Te^6+^O_6_ ν1 symmetric stretching
~640	Te^6+^O_6_ ν3 antisymmetric stretching	~640	Te^6+^O_6_ ν3 antisymmetric stretching
~665	α	~665	α
~685	Te^6+^O_6_ ν1 symmetric stretching	~685	Te^6+^O_6_ ν1 symmetric stretching
777	α	794	α
815	Si-O stretching		
821	Si-O stretching	821	Si-O stretching
945	metasilicate	945	metasilicate
974	Si-OH stretching	974	Si-OH stretching
1022	Si-O-Si bending	1022	Si-O-Si bending

^1^ Standard cubic cm per minute; ^2^ β-TeO_2_ phase; ^3^ α-TeO_2_ phase.

**Table 2:** Raman results for TZA thin films with 7 sccm oxygen flow and different annealing time.

7 sccm ^1^ 0 h		7 sccm 10 h	
110	AuTe_2_	110	AuTe_2_
137	AuTe_2_	133	AuTe_2_
152	AuTe_2_	150	AuTe_2_
166	AuTe_2_	166	AuTe_2_
229	α ^2^	229	Unidentifiable (noisy signal)
250	α	250	Unidentifiable (noisy signal)
300	α	300	Unidentifiable (noisy signal)
~380	Te^6+^O_6_ ν2 bending	~380	Unidentifiable (noisy signal)
415	α	415	Unidentifiable (noisy signal)
434	Zn-O bending	434	Unidentifiable (noisy signal)
~595	α	~595	Unidentifiable (noisy signal)
~618	Te^6+^O_6_ ν1 symmetric stretching	~618	Unidentifiable (noisy signal)
~640	Te^6+^O_6_ ν3 antisymmetric stretching	~640	Unidentifiable (noisy signal)
~665	α	~665	Unidentifiable (noisy signal)
~685	Te^6+^O_6_ ν1 symmetric stretching	~685	Unidentifiable (noisy signal)
813	Si-O stretching	813	Unidentifiable (noisy signal)
828	Si-O stretching	828	Unidentifiable (noisy signal)
945	metasilicate	945	Unidentifiable (noisy signal)
975	Si-OH stretching	975	Unidentifiable (noisy signal)
1021	Si-O-Si bending	1021	Unidentifiable (noisy signal)

^1^ Standard cubic cm per minute; ^2^ α-TeO_2_ phase.

**Table 3:** Interplanar distances results (d_spacing_) obtained from Figure 1b,d and the identified crystalline phases for 1 sccm oxygen flow and annealing time of 10 and 20 h.

d_spacing_ 10 h (Å)	d_spacing_ 20 h (Å ^1^)	Crystalline Phase			
3.24 ± 0.08					α-TeO_2_
	3.13 ± 0.08			ZnO	α-TeO_2_
2.98 ± 0.09	2.94 ± 0.07		AuTe_2_	ZnO	α-TeO_2_
	2.81 ± 0.07				α-TeO_2_
	2.71 ± 0.07			ZnO	α-TeO_2_
	2.44 ± 0.09			ZnO	
2.35 ± 0.08	2.33 ± 0.06	Au		ZnO	α-TeO_2_
2.1 ± 0.06	2.07 ± 0.05		AuTe_2_	ZnO	α-TeO_2_
	2.01 ± 0.05	Au			
	1.85 ± 0.03			ZnO	α-TeO_2_
1.72 ± 0.03	1.7 ± 0.03		AuTe_2_	ZnO	
	1.58 ± 0.02			ZnO	
	1.54 ± 0.02		AuTe_2_		
	1.48 ± 0.02			ZnO	
	1.43 ± 0.03	Au			
1.22 ± 0.02	1.23 ± 0.02	Au			
	1.18 ± 0.01	Au		ZnO	
	1 ± 0.01	Au		ZnO	
	0.932 ± 0.008	Au			
	0.908 ± 0.007	Au		ZnO	

^1^ Ångström (1 Å = 10^−10^ m).

**Figure A5:**
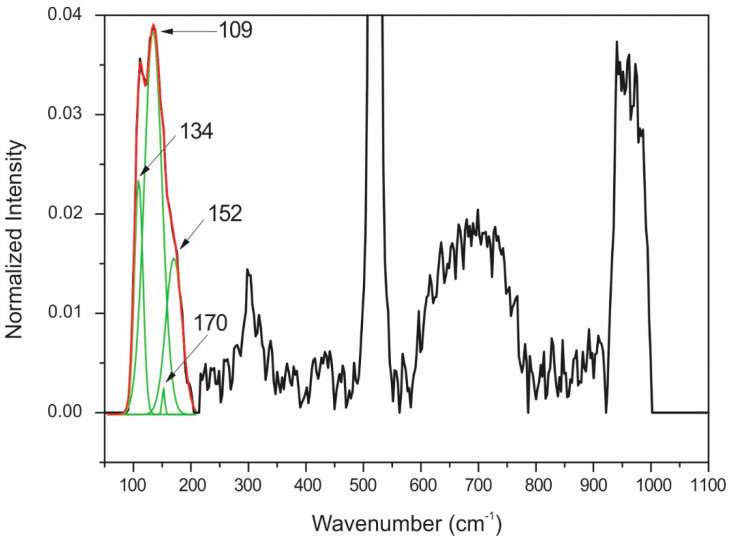
Measured Raman spectrum (black line), fitted gaussian bands (green line) and sum of the fitted gaussian bands (red line) for TZA thin films with process oxygen flow rate of 2 sccm posterior annealing at 325 °C in air for 10 h.

## Appendix A

Figure A1, Figure A2 and Figure A3 in the following show Raman results for TZ thin films with process oxygen flow rates of 0.5 sccm, 1 sccm, and 2 sccm, respectively, without posterior annealing. In addition, Figure A4 and Figure A5 show Raman results for TZA thin films with process oxygen flow rates of 1 sccm and 2 sccm, respectively, and posterior annealing at 325 °C in air for 10 h.
